# Advancement in fluid handling strategies for ultra-thin microfluidic devices

**DOI:** 10.1063/5.0344270

**Published:** 2026-08-04

**Authors:** Sarthak Saha, Sarah L. Perry

**Affiliations:** Department of Chemical and Biomolecular Engineering, University of Massachusetts Amherst, Amherst, Massachusetts 01003, USA

## Abstract

In this Perspective, we discuss the utility and flexibility of elastomeric pneumatic valving strategies and the challenges in applying these approaches to ultra-thin microfluidic devices. We use the strict material requirements for *in situ* x-ray analysis as a framework for introducing the challenges in leveraging pneumatic valves and highlight a range of capillary-based fluid handling and valving strategies that have been leveraged in x-ray applications as well as point-of-care diagnostics and biomedical applications where ultra-thin devices are critical. Rather than aiming to reproduce the full programmability of elastomeric valve networks, we highlight the ability of these platforms to facilitate event-based fluid control strategies that balance functional utility with manufacturability and robustness.

## INTRODUCTION

Since the inception of microfluidics, PDMS (polydimethylsiloxane) has played a critical role in device fabrication because of its transparency, biocompatibility, and ease of molding: key factors in creating intricate microchannel networks. A significant advancement in the field was the development of pneumatic valves that mimic the VLSI (very large-scale integration) architectures of microelectronics chips [Figs. 1(a)–1(d)].^1,2,64^ These elastomeric valves enable dynamic and precise control over fluid flow in microchannels, allowing for automated and accurate manipulation of liquids. The ability to create highly integrated systems with flow control, valving, and mixing functionalities has dramatically expanded the range of applications for microfluidic devices. PDMS-based microfluidic systems equipped with these valving mechanisms have been successfully deployed across a wide range of fields, including high-throughput screening for drug discovery,^3^ automated cell sorting,^4^ targeted drug delivery systems,^5^ and organ-on-a-chip platforms simulating physiological processes.^6^ The programmability and control over fluid dynamics using pneumatic valves in these systems have made PDMS microfluidic devices indispensable tools in both research and industry. With much of the research and development surrounding the fabrication and manufacturing of these valving systems now well established, PDMS-based microfluidic devices are primarily used today as sophisticated tools, and research efforts have largely transitioned to addressing application-specific needs.

At the same time, the very properties that make PDMS so powerful also constrain its broader applicability. PDMS is highly permeable to gases and readily absorbs small hydrophobic molecules,^7–10^ which can confound sensitive biological assays. It is also incompatible with many nonpolar solvents, and its hydrophobicity prevents straightforward capillary filling of channels with water. These drawbacks have driven researchers to explore alternative materials, such as more traditional hydrocarbon-based thermoplastics, polyimides, and newer classes of polymers, as well as paper—each tailored to specific applications. For example, paper-based microfluidic devices are widely used in point-of-care (POC) diagnostics, with common examples including diabetes test strips and pregnancy kits that rely on lateral flow-based assays.^11–16^ Thermoplastics, such as poly(methyl methacrylate) (PMMA),^17^ cyclic olefin copolymer (COC),^18–20^ and polycarbonate,^21^ are favored for their superior chemical resistance and mechanical stability. These materials also lend themselves to large-scale manufacturing, making them ideal for commercial applications in point-of-care devices and portable biosensors. Polyimides, known for their flexibility and thermal stability, are used in flexible electronics, wearable sensors, bioelectronics, and in the context of *in situ* x-ray analysis.^22–24^ Moreover, recent efforts have focused on developing novel photo-curable polymers like Norland Optical Adhesives (NOA),^25,26^ SU-8,^27,28^ and Ostemers,^29–32^ which tend to have better wetting properties for capillary-based devices.

However, this shift toward non-elastomeric and ultra-thin platforms introduces a fundamental challenge. Most alternative materials do not support membrane-based pneumatic valving and, therefore, cannot readily implement the programmability, addressability, and real-time actuation that define PDMS systems. Consequently, many thin, polymeric microfluidic devices rely on passive flow control or simplified actuation strategies that sacrifice functionality in exchange for reduced thickness and ancillary burden. This trade-off between device thinness and fluidic control now represents a central tension in the field. At its core, this tension reflects the lack of valving paradigms that are inherently compatible with ultra-thin, non-elastomeric microfluidic architectures.

The central premise of this Perspective is that the transition from elastomeric to ultra-thin microfluidics is not merely a material substitution, but a shift that fundamentally challenges existing fluidic control architectures. Traditional PDMS valving relies explicitly on multilayer devices with structural depth, elasticity, and membrane deflection to function. By eliminating these physical properties to achieve ultra-thin form factors, the field leaves a critical void in flow-control capabilities. Consequently, researchers are forced to rethink valving entirely, transitioning from architectures that mimic complex microelectronics to those that leverage the physical constraints of thin materials. This Perspective seeks to explore this paradigm shift, examining how the loss of traditional programmability has catalyzed the development of event-based fluid-handling strategies.

To frame this paradigm shift, it is necessary to explicitly define “event-based control” in contrast to existing fluidic strategies. Traditional PDMS pneumatic valves offer fully programmable, reversible, and continuous modulation of fluid flow, mimicking the architectures of microelectronics chips. Conversely, purely passive control, such as capillary flow in paper-based lateral-flow assays, relies on preprogrammed channel geometries and offers little adaptability or user-triggered control once a test is initiated. Event-based control occupies the critical space between these extremes. We define it as the execution of discrete, user-initiated fluidic operations, such as the localized removal of a hydrophobic barrier via a simple electrical trigger. While these events are often single-use and irreversible, they provide on-demand actuation without the structural and ancillary burden of pneumatic systems, offering a sufficient and highly practical level of functionality for ultra-thin platforms.

In this Perspective, we will explore innovative fluid-handling strategies for microfluidic devices fabricated from alternative materials, with a particular focus on ultra-thin devices. We begin our discussion with *in situ* x-ray analysis as its strict, well-defined material limitations provide a clear framework for understanding these valve challenges. Using this as a foundational example, we then broaden our scope to highlight how these strategies translate to the vastly more diverse and complex requirements of point-of-care diagnostics and biomedical applications, ultimately providing a perspective on the future directions of the field.

## VALVING CONSTRAINTS IN X-RAY COMPATIBLE MICROFLUIDICS

Over the last decade, significant progress has been made in the development of microfluidic devices for x-ray data collection, particularly for small-angle x-ray scattering and x-ray crystallography.^24,25,27,28,33–37^ While x-ray crystallography is used broadly to determine atomic structure, the difficulty in growing crystals of proteins and other biomacromolecules and the sensitivity of those crystals to handling and changes in environmental conditions are particular challenges.^38–40^ Microfluidic platforms have become powerful tools for protein crystallography, particularly for *in situ* and room temperature measurements that reduce crystal handling, maintain crystal quality, and better preserve native protein conformations.^24,27,36,37^

A significant challenge in designing devices for use with x rays is the need to limit signal attenuation, a factor that scales with the atomic number, density, and thickness of the materials present. While ultra-thin mixing devices for solution-phase x-ray scattering experiments can be fabricated out of hard materials like silicon,^41,42^ and do not require valving, crystallography platforms benefit from the ability to prepare samples with distinct compositions and were one of the first examples of high-throughput testing that leveraged pneumatic PDMS valves.^43^ However, the intrinsic silicon content of PDMS results in significant signal attenuation, motivating a transition to alternative materials for x-ray applications. To improve x-ray compatibility, efforts shifted toward fabricating microfluidic devices entirely from lower atomic number, carbon-based polymers, such as COC,^24,44^ PMMA,^27,28,45^ SU-8,^27,28^ and NOA.^25^ These materials significantly improved x-ray transparency, reducing signal loss and enhancing data quality. However, the transition to these non-elastomeric materials also exposed a persistent limitation: the loss of integrated, active fluid control. While hybrid platforms that combined thin layers of PDMS valves with carbon-based polymers could partially mitigate this issue,^36^ the achievement came at the cost of increased device complexity and reduced compatibility with streamlined experimental workflows.

More recent efforts in ultra-thin, polymer-based devices for *in situ* x-ray analysis have tended to prioritize signal transmission by eliminating PDMS and reducing material thickness to the greatest extent possible—in some instances using only atomically thin layers of graphene.^35,46^ In these systems, fluid handling is often driven by capillary forces, which naturally support filling and transport without external pumps [Fig. 1(e)]. It is also possible to introduce valves that similarly leverage capillary pinning, via a physical constriction and/or local surface treatment [Fig. 1(f)]. However, an important question then becomes the subsequent strategy to “open” valves and allow flow when desired.

One strategy for “opening” capillary valves has been the application of centrifugal force [Fig. 1(g)]. This strategy typically requires that the device be designed in a radial fashion such that both the rate of device rotation and the radial location of a given valve can be used to selectively overcome the capillary barrier to wetting at a given valve location. Originally pioneered for point-of-care and biomedical assays to eliminate the need for cumbersome fluidic ancillaries,^47,48^ this approach was subsequently adapted for structural biology workflows. Centrifugal valving has been used for protein crystallization,^49^ as well as *in situ* x-ray crystallography,^27^ and small-angle x-ray scattering.^50^ This strategy has the benefit of eliminating the need for external pressure pumps and tubing connections while enabling discrete control of events in an otherwise passive system. While such systems lack real-time programmability or multiplexed valve addressability, they offer a compelling trade-off between control and simplicity that is well matched to crystallization workflows and other structured experimental sequences. However, centrifugal architectures are inherently difficult to reconcile with standard rectilinear well-plate formats, and the requirement for continuous rotation complicates real-time observation. Moreover, while fluidic pumps are avoided, they are effectively replaced by bulky spinning hardware, limiting integration with imaging platforms and compact experimental setups.

An alternative strategy for valve actuation, with conceptual parallels to the programmable elements in VLSI systems, is electrical actuation. In microfluidic devices, electrical control of liquid motion has most commonly been implemented through electro-wetting on dielectric (EWOD),^51^ in which an applied electric field reversibly modifies the solid–liquid interfacial energy, reducing the contact angle according to the Young–Lippmann relation. While powerful, EWOD typically requires operating voltages on the order of tens of volts, above the electrolysis potential of water, limiting its compatibility with sensitive biological samples. In contrast, recent works have demonstrated electrically actuated valves that operate through a distinct, low-voltage mechanism^52,53^ suitable for sensitive biological samples.^54^ In these systems, a hydrophobically modified electrode acts as a capillary barrier until the application of a small potential (<1 V) irreversibly alters the surface chemistry, converting the valve region to a hydrophilic state and allowing flow to proceed [Fig. 1(h)]. Although the macroscopic outcome resembles EWOD, the underlying physics is fundamentally different, relying on electrochemically driven surface modification rather than reversible electrostatic wetting. This single-use, low-power actuation mode provides precise, localized control in material-constrained device architectures where pneumatic valves are impractical, reinforcing a broader shift toward event-based fluidic control strategies.

The significance of this electrically actuated approach lies not in replicating elastomeric valve functionality, but in providing precise, localized, and low-power control in device formats where pneumatic actuation is untenable. Crucially, this low-power requirement serves distinct needs depending on the application: for biological samples such as those present in protein crystallography, it avoids localized heating or electrochemical degradation of delicate samples, while for point-of-care diagnostics, it minimizes the reliance on bulky ancillary power supplies. Ultimately, utilizing simple, portable power sources opens new possibilities for integrating ultra-thin microfluidic devices with electronics, automation, and remote operation.

Despite the conceptual elegance of these alternative valving strategies, significant barriers to industrialization and standardization remain due to the inherent vulnerabilities specific to each approach. For instance, centrifugal microfluidics have struggled to achieve widespread adoption in highly automated laboratories because their requisite radial designs are fundamentally incompatible with industry-standard rectilinear well-plate formats and robotic liquid handlers. Similarly, EWOD systems, while versatile, frequently suffer from long-term reliability issues such as dielectric breakdown, biofouling, and droplet pinning, which complicate mass manufacturing. Purely passive capillary systems, while incredibly low cost, are notoriously sensitive to environmental conditions; slight fluctuations in ambient humidity or surface oxidation during storage can drastically alter contact angles and cause catastrophic device failure. Furthermore, the broader adoption of low-voltage electrically actuated valves must address their fundamentally single-use, irreversible nature, which precludes their use in complex fluidic routing that requires repeated opening and closing of the same channel. Because their actuation relies on an electrochemically driven surface modification, system performance is also heavily dependent on electrode-sample compatibility. Additionally, the stability of the hydrophobic patches prior to actuation, alongside the fabrication reproducibility required to scale these valves into highly multiplexed arrays, remains an ongoing challenge. Moving forward, transitioning these technologies from academic proofs-of-concept to standardized industrial tools will require addressing these specific material, operational, and manufacturing vulnerabilities.

Together, centrifugal and electrically actuated valving strategies exemplify a broader shift in microfluidic design philosophy (Table I): away from continuous, fully programmable control and toward event-based control architectures that are compatible with thin, rigid materials. While these approaches may be limited in complexity, they are well suited for crystallization workflows, reagent triggering, and time-resolved x-ray studies where control requirements are discrete and predefined. More broadly, they illustrate how embracing material constraints can yield new forms of fluidic control that expand the applicability of microfluidics beyond traditional PDMS platforms.

## PARALLELS IN POINT-OF-CARE AND BIOMEDICAL DEVICES

Notably, the design constraints that forced re-thinking of valving in x-ray compatible microfluidics, thinness, rigidity, limited ancillaries, and strict material requirements are nearly identical to those governing point-of-care and biomedical devices. In these settings, utilizing materials like PDMS with high air and water permeability is often a critical limitation as it can result in the evaporation of microscopic sample volumes over time. Further, point-of-care diagnostics prioritize affordability, portability, and ease of use, often favoring thin, disposable devices made from paper or plastic substrates. Paper-based microfluidics, which underpin widely used lateral-flow assays such as pregnancy and glucose tests, exemplify this approach. These systems rely on capillary flow and pre-programmed channel geometries to execute simple assays without pumps or valves. While highly successful, their functionality is limited by the absence of active control over fluid timing and routing.

Efforts to expand the capabilities of point-of-care devices have explored passive strategies such as geometric flow resistances, dissolvable barriers, and surface treatments to introduce delays or sequencing.^55–57^ Origami-style folding has further increased architectural complexity while preserving simplicity and low cost.^60–63^ More recently, capillaric circuits have emerged as a powerful framework for achieving autonomous fluid sequencing in thermoplastic devices without external pumps. These systems employ retention burst valves, trigger valves, and capillary pumping elements to execute multi-step assays through pre-programmed fluidic architectures.^58,65^ Such systems demonstrate that increasingly sophisticated assay workflows can be achieved through carefully engineered capillary networks rather than continuously actuated valves. While these approaches expand what can be achieved within strictly passive systems and illustrate an important shift away from purely geometric fluid programming and toward discrete, workflow-specific operations that are closely aligned with the requirements of many diagnostic assays, they offer little adaptability once a test is initiated and provide no mechanism for user-triggered or programmable control. As a result, there has been growing interest in incorporating minimal, event-based valving strategies into plastic point-of-care devices fabricated from thermoplastics such as PMMA, COC, polycarbonate, and polyethylene terephthalate (PET). These materials support thin, rigid device architectures compatible with scalable manufacturing, closely mirroring the physical constraints encountered in ultra-thin x-ray microfluidic platforms. Rather than adopting elastomeric pneumatic valves, control strategies developed for these systems draw on the same design philosophy described above: leveraging discrete triggering mechanisms that introduce control only where and when it is functionally necessary.

The viability of event-based control in these disparate fields becomes evident when comparing their specific operational requirements. In structural biology workflows, such as protein crystallization and *in situ* x-ray crystallography, the primary fluidic operations involve sample loading, reagent exchange, and time-resolved reagent triggering. These operations are inherently discrete, singular, and unidirectional. Similarly, point-of-care (POC) devices typically require operations such as sample metering, targeted reagent routing, isolation, and delayed reagent release. Because the required control in both fields is workflow-specific rather than continuous, simple event-based operations such as delayed reagent release or one-time sample isolation are often sufficient for successful assay execution. In this context, electrically triggered and centrifugally driven valves have emerged as particularly attractive approaches for point-of-care systems. The extreme versatility of electrical triggering is perhaps best demonstrated by its successful integration into ultra-low-cost, paper-based microfluidic platforms.^52,66^ Crucially, their appeal lies not in continuous programmability, but in their ability to enable single-step or workflow-specific control events such as delayed release and reagent routing or isolation using minimal infrastructure. These strategies embrace irreversibility and limited addressability as acceptable trade-offs for reduced system complexity, thinner form factors, and improved portability.

Despite the advantages of these fluidic control architectures, transitioning ultra-thin point-of-care devices into reliable commercial products requires addressing significant vulnerabilities regarding long-term reagent storage. Practical point-of-care systems rely heavily on preloaded, lyophilized bioactive reagents, such as enzymes, antibodies, and specialized buffers, which are acutely sensitive to humidity and water vapor permeation. Unlike conventional diagnostics that utilize thick, robust plastic casings, the thin polymer films or paper substrates characteristic of ultra-thin devices tend to exhibit high moisture vapor transmission rates and interface sealing instabilities. Consequently, these devices are highly prone to moisture penetration, which can prematurely rehydrate and degrade embedded biologicals, drastically reducing shelf life. Addressing this drawback will likely require the development of ultra-barrier vapor coatings or hybrid packaging that can protect sensitive reagents without compromising the ultra-thin form factor of the device.

While addressing these environmental vulnerabilities is critical for commercial viability, the underlying approach to fluid handling on these platforms has already undergone significant transformation. Across biomedical, point-of-care, and more domain-specific applications like x-ray compatible microfluidics, the challenge of valving in ultra-thin systems has driven a shift away from elastomeric, continuously actuatable control toward event-based valving architectures that are tightly matched to material and application constraints. This convergence emphasizes that the limitations encountered in ultra-thin microfluidics are not application-specific but instead reflect a broader redefinition of what constitutes meaningful fluidic control.

## OUTLOOK: FROM EVENT-BASED CONTROL TOWARDS PROGRAMMABILITY

The convergence of ultra-thin microfluidic device technology around centrifugal- and electrically triggered valving strategies highlights a broader shift in device design philosophy. Across a range of different applications, the material and form-factor constraints of ultra-thin devices, rigid substrates, and minimal ancillary infrastructure have necessitated a departure from continuous, pneumatic actuation toward event-based control paradigms. These approaches prioritize discrete, well-timed fluidic operations over full programmability, offering a practical path forward for applications where thickness, portability, and accessibility are paramount.

As microfluidic devices continue to become thinner, more application-specific, and more widely deployable, the definition of fluidic control must evolve accordingly. PDMS pneumatic valves remain unparalleled in versatility and multiplexed programmability, but they are increasingly misaligned with the physical and practical constraints of ultra-thin platforms. Rather than viewing alternative valving strategies as incomplete substitutes, they should be recognized as sufficient control architectures, which are optimized to deliver the level of control required to enable a given workflow while dramatically reducing device complexity and ancillary burden.

Looking forward, low-voltage electrically actuated valving represents a particularly promising direction for expanding the functional space of ultra-thin microfluidics. As these approaches mature, they offer a realistic pathway toward increasing control and complexity. Though such approaches may not fully replicate certain aspects of pneumatic valving, they remain compatible with thin, rigid materials and minimal power requirements. The fact that such systems can operate using simple power sources, including small batteries, positions them uniquely for both low-resource point-of-care settings and specialized biological applications such as *in situ* and time-resolved x-ray studies. Building on this, moving beyond simple event-based triggers toward “limited programmability” in ultra-thin devices will require innovative integration strategies. Because low-voltage electrically actuated systems can operate using these simple power sources, they are uniquely positioned to be paired with inexpensive micro-controllers or printed electronics. This could enable electrically addressable valve arrays that execute preprogrammed, timed sequences of fluid release without user intervention.

Rather than aiming to reproduce the full programmability of elastomeric valve networks, future progress is likely to involve selective increases in addressability, sequencing, and triggering within otherwise simple architectures. Incremental advances in electrically actuated control, combined with judicious use of centrifugal or passive elements, could enable hybrid control schemes that balance functional richness with manufacturability and robustness. By combining the strengths of different domains to create hybrid schemes, like integrating sequential electrical triggers with passive geometric flow resistances or combining low-power electrical routing with bulk fluid movement driven by centrifugal forces, could facilitate complex, multi-step assays. In this way, the field may move closer to the operational flexibility of pneumatic systems without inheriting their infrastructural complexity. By carefully matching simple, battery-powered triggering logic with structured spatial layouts, researchers can achieve a highly functional level of operational flexibility while avoiding the infrastructural complexity of traditional pneumatic networks.

Ultimately, the challenge and opportunity of any device and/or process design lies in identifying which control functions are truly necessary and implementing them in the simplest possible manner. Discrete valving events, irreversible switches, or one-time triggers may be entirely sufficient for crystallization, diagnostics, and sensing applications, while enabling ultra-thin microfluidic systems that are not only scientifically powerful, but also portable, affordable, and broadly accessible. By embracing sufficiency over maximal programmability, microfluidics can continue to expand its impact beyond specialized laboratories and into real-world settings where thin, robust, and low-power devices are essential.

## Figures and Tables

**FIG. 1.**
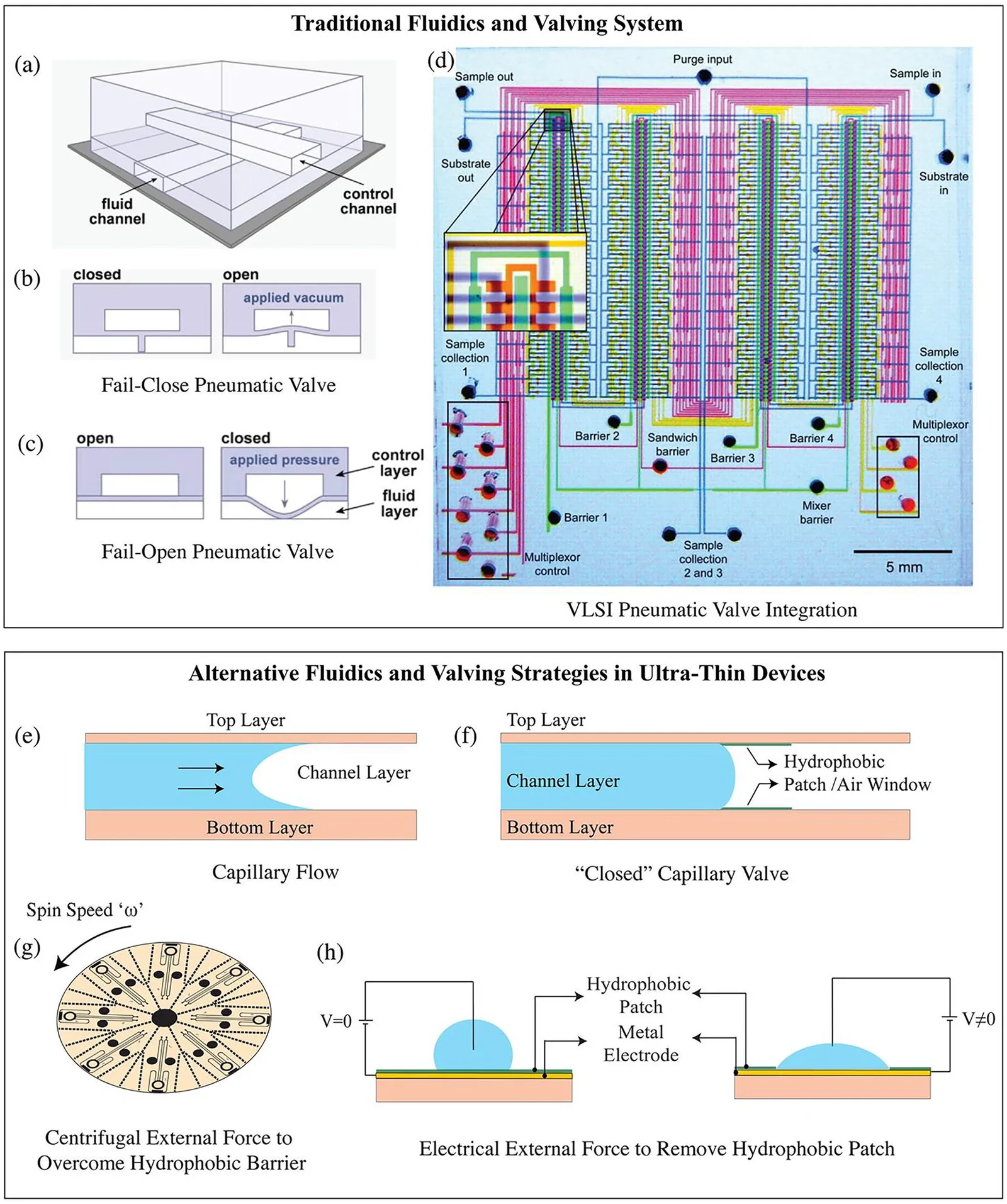
Overview of fluidic and valving strategies for traditional and ultra-thin microfluidic systems. (a) Schematic of a traditional multilayer PDMS microfluidic device showing independent fluid and control channels. (b) Fail-close pneumatic valve architecture, in which the applied vacuum deflects the control layer to open the flow channel. (c) Fail-open pneumatic valve architecture, where applied pressure deflects the control layer to close the flow channel. (d) Example of large-scale (VLSI) pneumatic valve integration enabling complex fluid routing through multiplexed control. Figure adapted from Thorsen *et al*., Science **298**, 580–584 (2002). Copyright 2002 AAAS.^64^ (e) Capillary-driven flow in ultra-thin, laminated devices, where liquid transport is governed by surface tension and contact angle. (f) A capillary valve in the closed state, in which a hydrophobic patch or air window prevents spontaneous flow. (g) Centrifugal valving, where increasing rotational speed generates an external force sufficient to overcome a hydrophobic barrier and initiate flow. (h) Electrically actuated valving, in which an applied potential removes the hydrophobic barrier to enable fluid movement.

**TABLE I.** Comparison of microfluidic valving strategies.

Valving techniques	Compatible materials	Actuation mechanism	Reversibility	Required equipment	Compatibility and issues
PDMS pneumatic valving^2,36,37,64^	Elastomers (PDMS)	Pressure/vacuum deflection of membrane	Reversible	External pressure pumps, tubing	Poor x-ray compatibility; high gas permeability can dry samples.
Capillary flow^15,55–59^	Paper, NOA, SU-8, PMMA, COC	Surface tension & contact angle	Irreversible	None	Good biocompatibility; no active flow control.
Centrifugal valving^27,50,60^	Thermoplastics, rigid polymers	Rotational force overcoming hydrophobic barrier	Irreversible	Bulky spinning hardware (centrifuge)	Good biocompatibility; poorly matched to rectilinear well-plate formats.
EWOD (electrowetting)^51^	Various substrates with dielectrics	Reversible electrostatic wetting	Reversible	High voltage power supply (>50 V)	High voltages can be incompatible with sensitive biological samples.
Low-voltage electrical valving^52,54^	Thin polymers (NOA, SU-8, etc.)	Electrochemical removal of hydrophobic barrier	Irreversible	Simple portable power sources (e.g., AAA batteries)	Good biocompatibility; allows only event-based fluid control.

## Data Availability

Data sharing is not applicable to this article as no new data were created or analyzed in this study.
